# Strengths–Weaknesses–Opportunities–Threats Analysis for a Pediatric Anesthesia Program

**DOI:** 10.1097/pq9.0000000000000254

**Published:** 2020-01-22

**Authors:** Ramón Eizaga Rebollar, María Victoria García Palacios, María del Carmen Fernández Mangas, Francisco Javier Arroyo Fernández, Carlos Miguel Márquez Rodríguez, Ana Isabel Carnota Martín, Javier Morales Guerrero, Luis Miguel Torres Morera

**Affiliations:** From the * Department of Anesthesiology and Reanimation, Puerta de Mar University Hospital, Cádiz, Spain; † Department of Preventive Medicine and Public Health, Puerta del mar University Hospital, Cádiz, Spain.

## Abstract

Risk management in healthcare institutions begins by first identifying the potential risks within a certain organization or specific area and then goes on to develop further strategies to reduce harm. The most common tool for this type of analysis is Strengths–Weaknesses–Opportunities–Threats (SWOT).

**Methods:**

We conducted a SWOT analysis in our pediatric anesthesia program: key factors were identified in a matrix, prioritized in a score table, represented in a graph, and finally analyzed.

**Results:**

Items obtained partial scores from 20 to 120. The item “lack of clinical protocols” was given greater weight (60) and received a lower value (1), resulting in the highest partial score (60) among the negative key factors and indicating a need for greater efforts to improve this specific aspect.

**Conclusion:**

The SWOT tool proved effective in identifying safety and quality key factors, and it provided information for initiating an improvement program.

## INTRODUCTION

Risk management in healthcare institutions combines the identification, assessment, and prioritization of potential risks to patients, to prevent adverse events occurrences in the future, or to minimize their consequences. This process begins by first understanding and identifying the potential risks within a certain organization or specific area and then goes on to develop strategies to overcome the barriers to program implementation.^1–3^

The most common tool for this type of analysis is Strengths–Weaknesses–Opportunities–Threats (SWOT), an acronym for the 4 parameters that this technique examines: Strengths, Weaknesses, Opportunities, and Threats.^4–6^ In the current literature, SWOT’s use in healthcare has been principally confined to identifying the key factors, arranging them in a matrix, and reflecting on them to develop an improvement plan or strategy.^1–6^ However, we consider that there are deficiencies in these past studies, as neither the SWOT analysis itself (with weights, values, partial scores for each item, and total scores for each key factor) nor the SWOT graph (with coordinates and the vector in the Cartesian plane) has been fully completed.

Over the last decade, there have been significant developments in our pediatric anesthesia program in various aspects such as clinical management, resident education, and scientific research. However, safety remains an issue that requires further work. Patient safety has undergone important advances in the last 20 years and should be a priority in current clinical practice, especially in the field of pediatric anesthesia. For this reason, we decided to start a risk management cycle, undertaking a SWOT analysis in our pediatric anesthesia program.

The objectives of this work are to address the safety issues and quality weaknesses, to identify opportunities for strategic program improvement, and to detect barriers to future risk management. These objectives are in line with our institution’s mission, vision, and values of (1) “satisfying patient needs and expectations through a safe, effective, and efficient practice, and promoting scientific production and continuous learning among professionals”; (2) “improving the quality and safety of patient care based on scientific evidence”; (3) “encouraging professional developing in care, learning, and research while ensuring that patients are at the center of the healthcare system, respecting their beliefs, culture, autonomy, privacy, and rights with a healthcare based on continuous quality improvement.”

## METHODS

To better understand the present situation with regards to patient safety in the pediatric anesthesia program of our hospital, we initiated a risk management cycle and conducted a SWOT analysis.

**Fig. 1.**
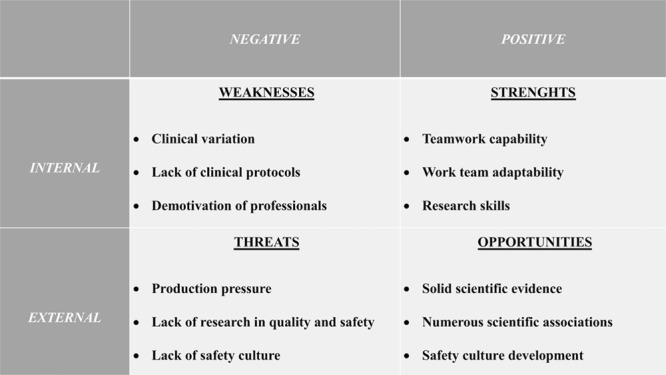
SWOT matrix. Internal/external and negative/positive key factors and items identified.

**Fig. 2.**
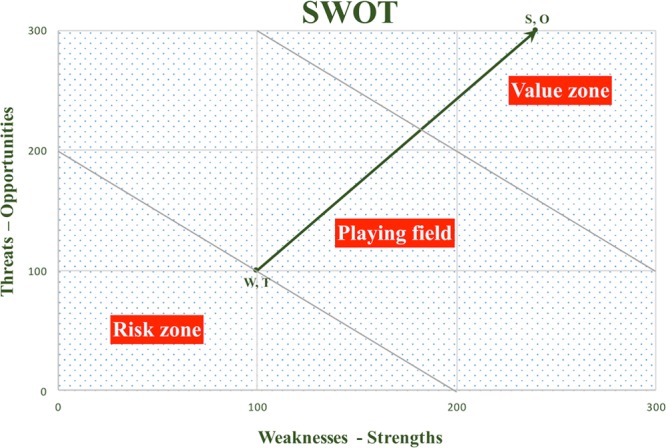
SWOT graph. The SWOT vector moved from the negative to the positive coordinate.

The steps taken were the following^7^:

- SWOT matrix: We identified the program’s internal factors (Weaknesses and Strengths) and external factors (Threats and Opportunities), arranging them in a matrix (Fig. 1).
- SWOT analysis: The identified items were prioritized or weighted by using the cumulative voting technique, also known as the “hundred-dollar method”: each key factor (S-W-O-T) was given 100 points, which could be distributed across the items according to its importance within each group. Likewise, each item received a value on a 4-category verbal rating scale (0 = very bad, 1 = bad, 2 = good, 3 = very good) according to its assessment at that time. Each item’s weight was then multiplied by its value to obtain a partial score. Finally, the sum of all partial scores resulted in the total score of each group or key factor (Table 1).^8^
- SWOT graph: We represented the total scores in a Cartesian coordinate system, with the internal key factors in the *x* axis and external key factors in the *y* axis. These scores were grouped into 2 pairs: Weaknesses–Threats (negative factors) and Strengths–Opportunities (positive factors) to form 2 coordinates joined by a vector. The SWOT vector moved from the negative to the positive coordinate and could be located in 3 differentiated zones representing distinct situations and entailing separate approach strategies (Fig. 2):
  - Risk zone (on the lower left side) shows the program within its environment in a poor light and suggests that the current strategy needs to be modified.
  - Playing field (in the middle of the graph) reveals that the program meets the demands and suggests a continuous improvement in the current strategy.
  - Value zone (on the upper right side) indicates that the program provides added value and recommends a strengthening of the current program strategy.
- SWOT vector analysis: We identified the key factors (S-W-O-T) required to improve the position of the vector on the SWOT graph.
- New approach strategy: Based on these key findings, we proposed a specific plan of action designed to move the SWOT vector toward the upper right area on the graph.

**Table 1.**
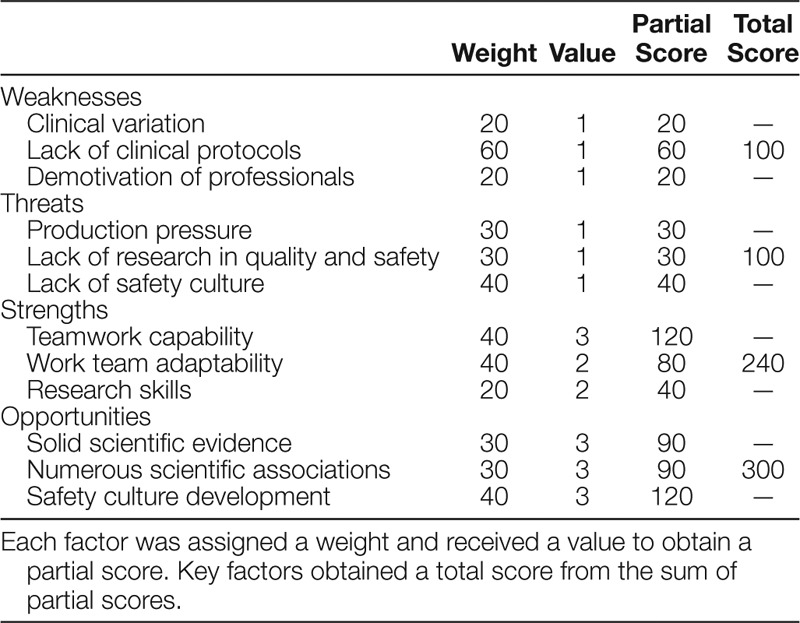
SWOT Analysis

## RESULTS

The “SWOT analysis” resulted in 4 total scores for each key factor, obtained from the weights and values assigned to every single item. These scores were 100 for Weaknesses and Threats, 240 for Strengths, and 300 for Opportunities (Table 1).

In the “SWOT graph,” total scores for Weaknesses (W), Threats (T), Strengths (S), and Opportunities (O) were represented on the Cartesian plane, providing 2 coordinates and a vector. We differentiated the negative pair (Weaknesses–Threats), whose coordinates are represented at the start of the vector, from the positive pair (Strengths–Opportunities), whose coordinates are represented at the end of the vector. This vector starts at the limit between the risk area and the playground area and ends inside the competitive advantage area (Fig. 2).

In the “SWOT vector analysis,” we identified and described the following items within each key factor and the significance of their vector position on the Cartesian plane.

### Weaknesses

We considered clinical variability as one of the major barriers to patient safety. This variability largely depends on the degree of clinical practice based on guidelines, pathways, and protocols. Hence, in this group, the item “lack of clinical protocols” was assigned the greatest weight (60/100), above the other 2 “clinical variability” and “demotivation of professionals,” which were assigned the same weight (20/100). All items received low values (1/3), so consequently, the total score of the key factor was also low (100/300), moving the initial point of the SWOT vector toward the left zone of the graph.

### Threats

We highlighted a lack of safety culture as a major barrier to patient safety. In our healthcare environment, safety culture is still in its early stages of development, with the implementing of frameworks based on safety models proving slow and difficult process, particularly when it comes to professionals training. A further threat which may hinder such implementation, and ultimately constitutes another important barrier to patient safety, is the production pressure. The development of a safety culture should be supported by solid scientific research to provide feedback throughout the implementation process and to promote continuous quality improvement research. Thus, in this group, the item “lack of safety culture” (40/100) was weighted above “production pressure” (30/100) and “lack of research in quality and safety” (30/100). As all items received low values (1/3), the key factor total score was also low (100/300), moving the initial point of the SWOT vector toward the lower zone in the graph.

### Strengths

We identified work capacity and adaptability as the most valuable internal qualities for a work team or program. The items ”teamwork capability” and “work team adaptability” were, therefore, equally weighted (40/100), above “research skills” (20/100). All of them received high (2/3) or very high values (3/3), and consequently, the overall key factor score was also high (240/300), moving the endpoint of the SWOT vector toward the right zone of the graph.

### Opportunities

We highlighted the advances in the development of safety culture in healthcare over the last 2 decades, with the creation of scientific associations and the publication of numerous research studies that form the basis for these advances. Hence, for this group, we decided to give similar weight to the items “safety culture development” (40/100), “solid scientific evidence” (30/100), and “numerous scientific associations” (30/100). They all received the maximum value (3/3), and this was reflected in the total score for this group (300/300), moving the terminal point of the SWOT vector toward the upper zone in the graph.

## DISCUSSION

A SWOT analysis serves as a preliminary decision-making tool that consists of a confrontation between internal capabilities and external developments of an organization to determine improvement strategies.^9^ The SWOT analysis of our pediatric anesthesia program resulted in a graph in which the vector moved from the upper limit of the risk zone to the competitive advantage zone, indicating the need to adopt a continuous improvement strategy to ensure patient safety (Fig. 2). After analyzing the SWOT vector, we determined that the key factors which required further work were the weaknesses. Within this group, the item “lack of clinical protocols“ received a greater weight and a lower value, resulting in the highest partial score among the negative key factors. Thus, this result indicates a need for greater efforts to improve this specific aspect (Table 1).

Once our analysis was complete, a reflection on its findings with regards to our pediatric anesthesia program ensued. On the one hand (internally), it was interesting to note that the identification of the degree of “clinical variability” was inversely related to the “lack of protocols” in our program. We observed some variability, but to a lesser degree than expected, given the limited number of clinical practice guidelines and protocols that we adapted to our program. Likewise, we identified “demotivation of professionals” as a dangerous item because it frequently appears to varying degrees and at different periods and impacts the rest of the key factors and the general functioning of any work team or program. In our environment, demotivation is associated with working conditions (ie, job situation, production pressure).

On the other hand (externally), the “teamwork capability” along with the “work team adaptability” proved noteworthy. This need to adapt to diverse situations was most evident with the complete turnover of the surgery team over the last 5 years and the substantial changes in the pediatric surgery program that this implies.

Finally, scientific production in our program has significantly increased in recent years and is presently probably our best weapon against clinical variability. From an external point of view, it is worth mentioning that the “production pressure” entails dedicating most time and effort to clinical practice to the detriment of research and specific training in safety. Moreover, it is important to mention the recent increase in safety culture development, with the creation of new scientific associations and the publication of numerous research articles that stress the importance of safety and quality in any health care organization.

Once we finished the SWOT analysis to assess the capabilities of our pediatric anesthesia program, the most relevant new information was the following: (1) among the internal key factors, the inversely proportional items “clinical variability” and “lack of clinical protocols” proved significant, particularly in the case of the latter, except when managing severe emergencies (ie, cardiac arrest, local anesthetic systemic toxicity, or malignant hyperthermia). (2) Among the external key factors, the “lack of safety culture” threat contrasted with the “safety culture development” opportunity because barriers preventing the development or enhancement of a safety culture in a particular program are still evident, despite recent advances in safety culture.

After conducting the SWOT analysis in our pediatric anesthesia program, other tools should be subsequently employed—within the risk management cycle—to assess the process risks and to assist in the decision-making process of corrective/preventive measures to reduce harm and to build up a safety culture, such as the Failure Modes and Effects Analysis and the Impact Effort Matrix.^10^

To the best of our knowledge, our study is innovative in that we have carried out a truly integral SWOT, with each step of the analysis fully completed. The resulting coordinate vector model represents an integral metric that accurately reflects the situational aspects of the program and, objectively, makes the SWOT analysis more accurate than a simple issue matrix. Thus, the main limitation of the SWOT analysis lies in the fact that both items and scores result from voting based on clinical experience and expert opinion. This approach may imply a certain degree of subjectivity and lack of prioritization or clarity of the issues involved.^11^

Looking to the future, we consider that this SWOT analysis could be the starting point for the implementation of a quality and safety strategy in our pediatric anesthesia program. Furthermore, we believe that this SWOT analysis could be adapted and conducted in other anesthesia programs to identify their own internal and external key factors, as an initial step before implementing a concrete improvement plan or safety and quality strategy.

In conclusion, the SWOT analysis conducted in our pediatric anesthesia program proved an effective tool to identify safety and quality weaknesses and opportunities and to provide information and arguments for initiating an improvement program within a quality and safety strategy.

## ACKNOWLEDGMENTS

I thank my sister Bárbara for supervising the English editing and for always being there for me.

## Disclosure:

The authors have no financial interest to declare in relation to the content of this article.
